# Strain Analysis in Patients with Frequent Premature Ventricular Complexes and Preserved Left Ventricular Function Undergoing Ablation

**DOI:** 10.3390/jcm12083017

**Published:** 2023-04-21

**Authors:** Sina Jamé, Zhigang Liu, Theodore Kolias, Jackson Liang, Troy Labounty, Michael Ghannam, Rakesh Latchamsetty, Krit Jongnarangsin, Fred Morady, Frank Bogun

**Affiliations:** Division of Cardiovascular Medicine, Cardiovascular Center, University of Michigan, SPC 5853, 1500 East Medical Center Drive, Ann Arbor, MI 48109, USA

**Keywords:** premature ventricular complexes, cardiomyopathy, ablation, left ventricular strain

## Abstract

Background: Frequent premature ventricular complexes (PVCs) can cause PVC-induced cardiomyopathy. The value of PVC ablation in patients with preserved left ventricular function in the low–normal range (ejection fraction: 50–55%) is not established. Strain analysis has been used to estimate changes in left ventricular function beyond assessment of the ejection fraction (EF). Longitudinal strain has been proposed as a method to detect changes over time in the setting of frequent asymptomatic premature ventricular complexes and preserved left ventricular (LV) function. A decrease in strain may be evidence of PVC-induced cardiomyopathy. Objective: In this study, we assessed the role of PVC ablation in patients with low–normal EF and the effect on EF and myocardial strain before and after PVC ablation. Methods: A total of 70 consecutive patients with either low–normal EF (0.5–<0.55, *n* = 35) or high–normal EF (≥0.55; *n* = 35), using available imaging and Holter data, were referred for ablation due to frequent PVCs. EF and longitudinal strain were assessed pre- and post-ablation. Results: There was a significant increase in EF (53.2 ± 0.4% to 58.3 ± 0.5%, *p* < 0.001) and improvement in longitudinal strain (−15.2 ± 3.3 to −16.6 ± 3, *p =* 0.007) post-ablation in patients with low–normal EF and successful ablation. There was no change in EF or longitudinal strain in patients with high–normal EF and a successful ablation pre- vs. post-ablation. Conclusions: Patients with frequent PVCs and low–normal LV EF compared to patients with frequent PVCs and high–normal LV EF have evidence of PVC-induced cardiomyopathy and may benefit from ablation despite a preserved left ventricular EF.

## 1. Background

Frequent premature ventricular complexes (PVC) can cause cardiomyopathy. A high PVC burden [1], long exposures to PVCs [2], asymptomatic status [2], epicardial PVC origins [3], broader PVC QRS complexes [3], the presence of interpolated PVCs [4], and the presence of scarring [5], among others, have been considered as factors associated with the development of PVC-induced cardiomyopathy. A left ventricular ejection fraction (EF) of <50% has been considered as a cut-off to distinguish patients with PVC-induced cardiomyopathy from patients with a preserved EF [1,2,4,6]. In patients presenting with frequent asymptomatic PVCs and preserved ejection fraction (EF) of ≥50%, a watchful waiting strategy has been recommended [7].

However, even in the presence of preserved left ventricular (LV) function, frequent PVCs may still impact on ventricular performance. Longitudinal strain has been proposed as a method to determine ventricular function beyond the EF. A decrease in longitudinal strain may precede a reduction in LV EF in the setting of chemotherapy [8,9,10]. Decreased longitudinal strain that increases after successful ablation has been described in patients with preserved LV EF and frequent PVCs [11].

Furthermore, an EF of >55% has also been considered to indicate normal function in the echocardiography literature [12]. The value of longitudinal strain in patients with a range of preserved ejection fractions (50–55% vs. >55%) and frequent PVCs has not been assessed. The purpose of this study was to assess the impact of PVC ablation in the presence of low–normal (50–55%) and high–normal (>55%) LV EF and to assess the change in EF and longitudinal strain before and after ablation.

## 2. Methods

### 2.1. Inclusion and Exclusion Criteria

The patients were divided into 2 groups: the group with a low–normal EF (range from 50–55%) and frequent PVCs and the control group, consisting of patients with high–normal EF (>55%) and frequent PVCs. Patients with an EF < 50% were excluded; patients with known non-ischemic cardiomyopathy or ischemic cardiomyopathy were also excluded.

### 2.2. Holter Recordings

A 24-h Holter was recorded prior to the ablation procedure. Frequent PVCs were defined as a PVC burden of >5%. The recording was repeated 3–4 months post-ablation. A decrease of ≥80% in the PVC burden was defined as a successful ablation of PVCs.

### 2.3. Mapping and Ablation

The study was approved by the University of Michigan Internal Review Board. After informed consent was obtained, several multipolar catheters were advanced and positioned at the His position and the right ventricular apex. A total of 3000 units of Heparin was administered and 1000 units of heparin were given every hour for right sided procedures and for left sided procedures; a target ACT of 250 s was used. A 3-D-electroanatomic mapping system (CARTO, Biosense Webster, Diamond Bar, CA, USA) with a 3.5 mm irrigated-tip catheter (Thermocool, Biosense Webster) was used. Activation mapping was used to identify the site of origin in the presence of frequent PVCs. If PVCs were infrequent during the procedure, pace mapping was used to map the origin of the PVCs. At the site of origin, radiofrequency energy was delivered with an initial power of 20 Watts that was increased up to 50 Watts to achieve an impedance drop of 10 Ohms. Radiofrequency energy was applied for 60–120 s. Surface electrograms were recorded in combination with bipolar intracardiac tracings and recorded on optical disc (Workmate Claris^TM^, Abbott Laboratories, Pleasanton, CA, USA).

### 2.4. Echocardiography

Echocardiograms were reviewed by three independent observers. In order for patients to qualify for inclusion in the low–normal EF group, at least 2 of 3 observers had to measure the EF by using Simpson’s formula to be 50–≤55%. The echocardiographers were blinded to the measurements of the other readers. The EF was determined by analyzing the second sinus beat after a PVC. Echocardiographic images were obtained prior to ablation and 3–4 months after ablation with available commercial equipment. Systolic function assessment was conducted retrospectively, using the biplane method of disks summation to calculate left ventricular ejection fraction (Synapse Cardiovascular PACS, 6.0.3) [13]. The endocardial borders were manually traced in the usual fashion from one aspect of the mitral annulus to the other for assessment, excluding trabeculations and papillary muscles.

### 2.5. Strain Analysis

**Figure 1:**
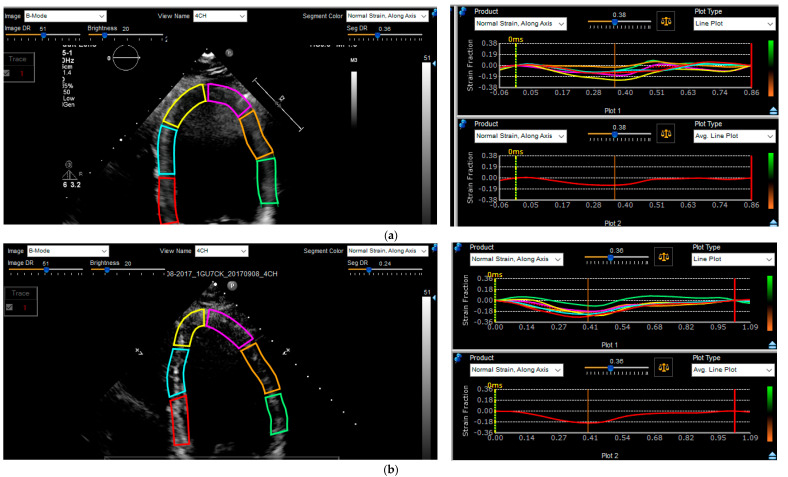
Comparison of pre- (**a**) to post- (**b**) PVC ablation strain analysis. Segmental contours are highlighted on the panel on the left. The longitudinal strain over time is visualized segmentally on the top right panel and globally on the bottom right panel. The global longitudinal strain was −10.7 prior to ablation (**a**) and it was −19.2 post-ablation (**b**).

The analysis was performed by two readers blinded to ablation outcomes (Figure 1); the strain measurements from the 2 readers were averaged. In summary, none foreshortened apical four chamber Digital Imaging and Communications in Medicine (DICOM) images were digitally stored at 30 frames/second for further longitudinal strain analysis with post-processing software (EchoInsight, version 3.1.03.3870, Epsilon Imaging, Ann Arbor, MI, USA). The LV border at the end of diastole was manually identified by the reader. The software then automatically tracked the LV boundary throughout the cardiac cycle, with further manual adjustments made as indicted. The software divided the LV into six segments and presented the data graphically (Figure 1). The peak systolic negative value of the averaged strain curves was then obtained. The four chamber view was solely used in this study, as prior studies have demonstrated high correlation with strain analysis using alternate views [14].

The inter-observer variability had an inter-observer mean error for the Epsilon strain analysis software of 8% and an intra-observer mean error of 7% [14].

### 2.6. Follow-Up

After a successful ablation, antiarrhythmic medications were discontinued. Patients were seen in follow-up 3–4 months post-ablation and 12 months thereafter. Holter monitoring and echocardiograms were repeated 3–4 months post-ablation.

### 2.7. Statistical Analysis

Continuous variables were expressed as mean ± 1 standard deviation and were compared with Student’s t-test. Categorical variables were compared with the Chi square test. If the sample size was smaller than 5 in a given cell, Fisher’s exact test was used. The Pearson correlation coefficient was used to correlate longitudinal strain with EF. Echocardiograms were read by at least 3 trained echocardiographers, who were blinded to the inclusion and exclusion criteria of the study. Furthermore, strain analysis was performed by 2 echocardiographers blinded to the ablation outcome.

A *p* value < 0.05 was considered statistically significant.

Statistical analysis was performed using R version 4.1.1 (R foundation for Statistical Computing, Vienna, Austria).

## 3. Results

### 3.1. Patient Characteristics

The study population was obtained through retrospective review of 174 consecutive patients with frequent PVCs. Of these patients, 35 patients met inclusion criteria of low–normal systolic ejection function (50–<55%) on a pre-ablation echocardiography, in the presence of frequent PVCs (17 women, 53.9 ± 15.7 years, mean EF of 53.3%) and in the absence of structural heart disease. These patients were compared to a control group of 35 consecutive patients with high–normal EF (>55%) and frequent PVCs (17 women, 58.7 ± 12.7 years, mean EF of 61.4%, Table 1). Two thirds of the patients were symptomatic with palpitations. Four patients had prior syncope and ten patients had prior failed ablations. Table 1 compares the characteristics of patients in the study group with the control group.

### 3.2. Ablation Details

Catheter ablation was successful in 55 patients (79%; 28 patients in the study and in 27 patients in the control group).

The site of origin of the PVCs was the RVOT (*n* = 19), the LVOT and sinuses of Valsalva (*n* = 12), the mitral annulus (*n* = 5), the epicardium in seven, intramural (basal septum) in nine, the papillary muscles in eight, and other sites in eleven patients.

In the study group the PVC burden was reduced from 21.8 ± 12% to 3.2 ± 6.3%, and in the control group the PVC burden was reduced from 22.4 ± 11% to 3.8 ± 7.9% (both *p* < 0.001).

The PVC burden was reduced from 23 ± 11% to 1.2 ± 1.8% in all patients with an effective ablation and from 17 ± 10% to 13 ± 12% (*p =* 0.04) in patients with an ineffective ablation.

### 3.3. Echocardiographic Analysis

The prevalence of atrial fibrillation and arterial hypertension, hyperlipidemia, diabetes mellitus, chronic renal insufficiency, chronic obstructive lung disease, and coronary artery disease was similar in patients with a low–normal EF compared to patients with normal ejection fraction (*p* > 0.05 for all comorbidities).

With a successful ablation, the EF improved from 53.2 ± 0.4% to 58.3 ± 0.5% in the study group (*p* < 0.001, Table 2) and remained similar in the control group (62 ± 0.4% vs. 62 ± 0.4%, *p =* 0.56). In those with a failed ablation, the baseline EF did not change following ablation in the study group (53.0 ± 0.6% to 55.6 ± 0.7%) or the control group (61.7 ± 0.4% to 62.7 ± 0.4%), which was *p* > 0.05 in both.

The baseline longitudinal strain in patients with low–normal EF was lower compared to patients with high–normal LVEF (−15.2 ± 3.3 vs. −17.4 ± 3, *p* < 0.001). More patients with low–normal EF had an abnormal longitudinal strain compared to patients with a high–normal EF (19/35 vs. 10/35, *p =* 0.02).

**Figure 2:**
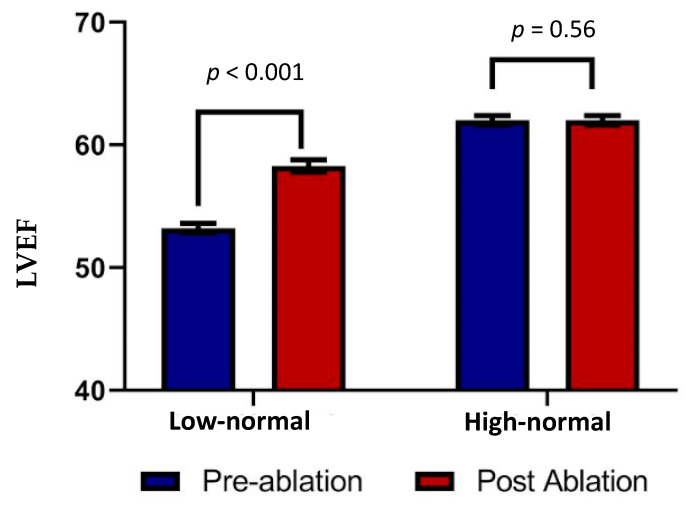
Comparison of change in LV systolic ejection fraction (EF) in patients with low–normal and high–normal EF following successful ablation.

The longitudinal strain improved from −16.5 ± 3.2 to −17.4 ± 3 in patients who had a successful ablation (*p =* 0.009, Figure 2). The longitudinal strain in patients with an ineffective ablation remained the same (−15.8 ± 4 vs. −16 ± 3, *p =* 0.8).

In patients with a low–normal EF (study group), the longitudinal strain improved from −15.2 ± 3.3 to −16.6 ± 3 (*p =* 0.007, Table 2) and remained unchanged in patients with a high–normal EF (control group, −17.4 ± 3 vs. −17.6 ± 3, *p =* 0.63, Table 2). In nine patients with an EF >55%, the strain was >−0.16. In these patients, six had successful and three had failed ablations. In patients with a successful ablation the strain improved from −12.9 ± 1.5 to −15 ± 2.4 (*p =* 0.09) and in three patients with ineffective ablations the strain remained unchanged (−13 ± 2.1 vs. −12.4 ± 3.4, *p =* 0.7).

In patients with improvement in EF, the preprocedural PVC burden was no different from patients without improvement in EF (23 ± 11% vs. 18 ± 10%, *p =* 0.2). The same is true for patients with improved strain post-ablation; their preprocedural PVC burden was not significantly different from patients without improvement in strain post-ablation (24 ± 10% vs. 20 ± 13%, *p =* 0.14). Additionally, when comparing the PVC origins (epicardial vs. non-epicardial origins), there was no difference in origins associated with cardiomyopathy in patients with improved EF vs. patients without improved EF post-ablation (six epicardial origins vs. zero epicardial origins; *p =* 0.6). Similarly, there was no difference in the prevalence of epicardial origins in patients with improved vs. non-improved strain post-ablation (four epicardial origins vs. two epicardial origins, *p =* 1).

There was a significant correlation between the pre-ablation EF and the pre-ablation strain (R = 0.41, *p =* 0.0004). There also was a significant correlation between post-ablation EF and post-ablation longitudinal strain (R = 0.31, *p =* 0.008).

There was no correlation between pre-ablation longitudinal strain and PVC burden (R = 0.06, *p =* 0.61).

### 3.4. Follow-Up

Antiarrhythmic medications were discontinued in all patients with a successful ablation procedure. In four patients, antiarrhythmic medications were continued due to a failed ablation. Four patients had recurrent PVCs during follow-up and required repeat ablations.

## 4. Discussion

### 4.1. Main Findings

Both ejection fraction and longitudinal strain improved after a successful ablation procedure in patients with a low–normal EF. Most patients with frequent PVCs and a low–normal EF had reduced longitudinal strain. These findings indicate that there is evidence of impaired left ventricular performance that improves after successful PVC ablation, even when the LV EF is in the normal range.

### 4.2. Left Ventricular Function and Frequent PVCs

Data reported from PVC ablation studies are often dichotomized to patients with an abnormal EF of <50% vs. patients with preserved EF of ≥50% [1,6]. In this study, we sought to analyze a patient population with EFs in the normal range (≥50%), focusing on patients with low–normal (EF 50–≤55%) vs. patients with high–normal EF (EF > 55%). Despite a low–normal ejection fraction, there was evidence of impaired longitudinal strain indicating abnormal left ventricular performance. Both EF and strain improved significantly with a successful ablation procedure. The study, therefore, provides evidence that even in patients with preserved EF, PVCs have an impact on left ventricular performance. On the other hand, strain was unaltered in patients with a normal EF and frequent PVCs when a successful ablation was carried out. Hence, the study supports strong consideration of an ablation procedure to suppress PVCs in the presence of a low–normal ejection fraction. Furthermore, our data support that, in the presence of normal LV function with an EF of ≥55%, an approach using a watchful waiting strategy may be reasonable. Longitudinal studies will be beneficial to determine whether a change of strain over time will help to identify patients who are more likely to benefit from ablation in the presence of normal EF of >55%.

A high PVC burden is associated with PVC-induced cardiomyopathy. Hence, it is interesting that longitudinal strain was not associated with the PVC burden. The PVC burden is not the only determinant of PVC-induced cardiomyopathy and not everybody with a high PVC burden will develop cardiomyopathy [1]. About 20% of the patients in the study by Baman et al., despite having a PVC burden of >24%, did not have cardiomyopathy by echocardiography. Factors other than the PVC burden are associated with the development of PVC-induced cardiomyopathy [2,3,4,5] and these factors are independent of the PVC burden associated with PVC-cardiomyopathy. Therefore, it is not surprising that the PVC burden did not correlate with strain and yet patients with abnormal strain did have improvement in the strain after a successful ablation, whereas patients with failed ablations did not. Lack of a correlation between PVC burden with longitudinal strain has also been reported by others [11,15,16].

### 4.3. Strain Analysis

Longitudinal strain has been used to detect subclinical left ventricular dysfunction. The association of a low–normal EF with abnormal strain as well as the improvement in EF and increase of strain after successful ablations supports the finding that, despite an EF within the normal range, the LV performance is impaired. This dysfunction is most likely due to PVC-induced cardiomyopathy. In a prior report, an abnormal strain was detected in patients with frequent PVCs and preserved EF that improved after successful ablation [11]. However, the EF in the latter study is not specified and it is unclear how many patients with a low–normal EF may have been included.

Based on the available literature, ventricular strain measurements are highly reproducible [17], with a low intra- and inter-observer variability. The data of the strain analysis corroborate the hypothesis that in patients with a low–normal EF, frequent PVCs have impacted on the LV performance, despite the EF being within the normal range.

### 4.4. Limitations

The inclusion criteria aimed to capture a patient population with a relatively narrow range of ejection fraction. Since inter-observer variability of assessment of the EF may be beyond the targeted range of EF, three rather than two observers read the baseline echocardiograms and at least two of the three readers had to agree with the EF range of 50–55%. The fact that the longitudinal strain was also abnormal in these patients supports the notion that the patients with low–normal EF were correctly identified. Furthermore, strain was assessed in a retrospective analysis of DICOM images with a frame rate of 30/s; prospective analysis is required to confirm the results of these findings. A common limitation of strain analysis is the lack of a normal reference because the values are dependent on the software that was used. This limits the generalization of our data.

## 5. Conclusions

Patients with frequent PVCs and low–normal LV EF compared to patients with frequent PVCs and high–normal LV EF have evidence of PVC-induced cardiomyopathy and may benefit from ablation despite a preserved left ventricular EF.

## Figures and Tables

**Table 1:** Patient characteristics.

Variable	Patients with EF 50–<55%	Patients with EF ≥ 55%	*p*-Value
Patients (*n*)	35	35	
Age	53.9	58.7	0.17
Sex (Male %)	18 (51.4)	18 (51.4)	1
EF (%)	53.3	61.4	<0.001
PVC Burden (%)	21.7	21.7	0.82
**Comorbidities**			
DM (%)	6 (17.1)	6 (17.1)	1
HTN (%)	14 (40.0)	17 (48.6)	0.47
AF (%)	2 (5.7)	2 (5.7)	1
COPD (%)	1 (2.9)	3 (8.6)	0.31
HLP (%)	12 (34.3)	18 (51.4)	0.15
Renal Insufficiency (Cr >1.5)	1 (2.9)	3 (8.6)	0.31
OSA (%)	7 (20.0)	10 (28.6)	0.41
CVA (%)	2 (5.7)	2 (5.7)	1
**Medical Therapy**			
Beta-Blocker	25 (71.4)	23 (65.7)	0.61
Verapamil	8 (22.6)	3 (8.6)	0.10
Diltiazem	2 (6.0)	5 (14.3)	0.24
Digoxin	0	0	1
ACEI/ARB	10 (28.6)	11 (31.4)	0.80
AAD	11 (31.4)	9 (26.0)	0.60
Prior Ablation Procedure	6 (17)	4 (11)	0.50

Abbreviations: EF = ejection fraction, PVC = premature ventricular complex, DM = diabetes mellitus, HTN = hypertension, AF = atrial fibrillation, COPD = chronic obstructive pulmonary disease, HLP = hyperlipidemia, OSA = obstructive sleep apnea, CVA = cerebrovascular accident, ACE/ARB = angiotensin converting enzyme inhibitor/angiotensin receptor blocker, AAD = antiarrhythmic drug.

**Table 2:** Echocardiographic and Holter data pre- and post-ablation.

Variables	Before Ablation	Post-Ablation	*p*-Value
EF (%)			
- Low nl EF-Successful RF	53.2 ± 0.4	58.3 ± 0.5	<0.001
- Low nl EF-Failed RF	53 ± 0.6	55.6 ± 0.7	>0.05
- High nl EF-Successful RF	62 ± 0.4	62 ± 0.4	0.56
- High nl EF-Failed RF	61.7 ± 0.4	62.7 ± 0.4	>0.05
PVC Burden (%)			
- Low nl EF	21.8 ± 12	3.2 ± 6.3	<0.001
- High nl EF	22.4 ± 11	3.8 ± 7.9	<0.001
Longitudinal Strain			
- Low nl EF-Successful RF	−15.2 ± 3.3	−16.6 ± 3	0.007
- Low nl EF-Failed RF	−15.8 ± 4	−16 ± 3	0.8
- High nl EF-Successful RF	−17.4 ± 3	−17.4 ± 3	0.63
- High nl EF-Failed RF	−19.1 ± 0	−19.0 ± 0	0.9

## Data Availability

The data of this study can be obtained upon reasonable request.
